# Psychometric Properties of Interpersonal Needs Questionnaire-15 for Predicting Suicidal Ideation among Migrant Industrial Workers in China

**DOI:** 10.3390/ijerph18147583

**Published:** 2021-07-16

**Authors:** Rongxi Wang, Yexin Chen, Fan Hu, Zixin Wang, Bolin Cao, Chen Xu, Xiaoyue Yu, Ruijie Chang, Huwen Wang, Hui Chen, Yujie Liu, Shangbin Liu, Tian Hu, Yaqi Chen, Kechun Zhang, Huachun Zou, Yong Cai

**Affiliations:** 1 School of Public Health, Shanghai Jiao Tong University School of Medicine, Shanghai 201800, China; RosieW816@outlook.com (R.W.); chenyexin888@sjtu.edu.cn (Y.C.); joyking2003@163.com (F.H.); xuchen233333@163.com (C.X.); dd2192003@163.com (X.Y.); 13916984965@yeah.net (R.C.); tanjaw@163.com (H.W.); chenhui711@yeah.net (H.C.); liuyj_4287@163.com (Y.L.); liushangbin12139@163.com (S.L.); 2 JC School of Public Health and Primary Care, Faculty of Medicine, The Chinese University of Hong Kong, Hong Kong 999077, China; wangzx@cuhk.edu.hk; 3 School of Media and Communication, Shenzhen University, Shenzhen 518000, China; caobolin@szu.edu.cn; 4 Shenzhen Longhua District Center for Disease Control and Prevention, Shenzhen 518000, China; ht1137571641@126.com (T.H.); chloe4697@163.com (Y.C.); 5 School of Public Health, Sun Yat-sen University, Panyu District, Guangzhou 511431, China; 6 Kirby Institute, University of New South Wales, Sydney 2052, Australia

**Keywords:** perceived burdensomeness, thwarted belongingness, social isolation, Interpersonal Needs Questionnaire, suicidal ideation, migrant workers

## Abstract

Objective: Interpersonal theories of suicide suggest that the Interpersonal Needs Questionnaire (INQ) can be used to measure suicidal ideation, but few studies have focused on migrant people, a group with a high prevalence of suicidal ideation. The aim of this study was to validate the psychometric properties of the INQ-15 and its prediction of suicidal ideation among migrant industrial workers in China. Method: A stratified multi-stage sample of 2023 industrial workers was recruited from 16 factories in Shenzhen, China. There were 1805 nonlocal workers, which we defined as migrant workers with a mean age of 32.50 ± 8.43 years old who were 67.3% male. The structure of the Chinese version of the INQ-15 and its correlation and predictive utility for suicidal ideation were examined through factor analysis, the Item Response Theory, the M2 test, logistic regression, and receiver operating characteristic (ROC) analysis. Results: Different from studies among various samples in which a two-factor solution is identified, results within this sample indicated three factors: perceived burdensomeness, thwarted belongingness, and social isolation. The model fit statistics of three-factor INQ were 0.075 for RMSEA, 0.945 for CFI, 0.932 for TLI, and 0.067 for SRMR. The model standard estimated factor loadings ranged from 0.366 to 0.869. The summed scores of INQ and perceived burdensomeness predicted suicidal ideation after controlling for sociodemographic characteristics such as age, gender, and income with AUC of 0.733 (95% CI: 0.712/0.754) and 0.786 (95% CI: 0.766/0.804). In the meantime, the comparison of the predictive ability between INQ total scores and PB scores was significant with *p* < 0.05. Conclusion: The INQ has good psychometric properties and can be used to assess how migrant workers living in the Shenzhen perceive meeting interpersonal psychological needs and shows good predictive ability of suicidal ideation. Perceived burdensomeness appears to play a role in suicide and may be a point of intervention, yet the notable deviation from previous findings and the relative weakness of two of the other factors warrant further study.

## 1. Background

With global economic integration, international and intranational migration is common [1]. Economic reform in China and the transition to a market economy resulting in massive internal migration as workers were allowed to move from rural areas to the economically productive areas. Eastern and coastal regions in the Pearl River Delta including Shenzhen were major destinations for migrants from impoverished western and central inland areas of China. The nationwide internal migrant population reached 0.26 billion in 2010 [2]. According to the National Seventh Population Census, approximately 70% of resident population in Shenzhen are mobile people [3]. Migrants are at the bottom of the social ladder and are discriminated against, have a high prevalence of mental health problems, and have experienced a high incidence of suicide [4]. Suicide accounts for approximately 800,000 global deaths each year and is a serious global public health problem that needs to be addressed [5]. The World Health Organization (WHO) estimates that the suicide rate in 2016 was 10.6 per 100,000 people, with 80% of these suicides occurring in low- and middle-income countries [6]. Death by suicide additionally produces a profound emotional impact on bereaved loved ones [7,8]. Understanding the causes of suicide, and best practices for assessing, preventing, and treating suicidal behavior, has been identified as a key public health priority and global imperative [9,10] The Interpersonal Theory of Suicide (IPTS) proposes that suicide occurs with two constructs, namely the acquired capacity for lethal self-injury and the desire to die, neither of which alone is sufficient to cause an individual to die by suicide [11,12]. The most dangerous form of suicidal ideation is caused by the simultaneous presence of two interpersonal constructs—thwarted belongingness (TB) and perceived burdensomeness (PB). TB describes the unmet need of being socially integrated, and PB reflects the perception of being a burden for others [13].

The Interpersonal Needs Questionnaire (INQ) was developed to assess the main constructs (TB and PB) of the IPTS for predicting suicidal desire [14]. The factor structure of the INQ has been validated by many researchers and among different populations [15,16,17,18,19,20,21,22]. However, results have been inconsistent, with most studies validating a better model fit for the two dimensions of the INQ [18,23,24,25,26], and some studies validating a better model fit for the three dimensions of the INQ [27]. Currently, most studies on suicide focus on students [28,29,30,31] and depressed patients [32,33,34], and few studies have been conducted on migrant industrial workers, whose population reached 174 million in 2019, which was an important feature of China’s economic development [1]. Surveys have shown that 14.7% of migrant workers are at high risk of mental illness and may perceive unmet interpersonal needs, which are considered proximate and causal factors for suicidal thoughts and behaviors [35]. One study estimated that from 2015 to 2017, the annual suicide rate among migrant workers in Zhongshan, China was 4.46/100,000, slightly higher than the city average (4.03/100,000) [36]. The successive incidents of suicides and deaths of migrant workers have drawn particular attention to the psychological conditions of young migrant workers on the production line [4]. In past studies, there have been several versions of the INQ, including 10-, 12-, 15-, and 25-item questionnaires [14,24,26,37,38], whereas Hill et al. recommended using the 15- or 10-item versions because these versions indicated the most consistent model fit in confirmatory factor analyses [39]. In this study, we primarily used the INQ-15 recommended by the Van Orden K.A. [14].

The aim of this study was to validate the reliability and validity of the INQ-15 among industrial workers in Shenzhen, China, thereby informing the application of the INQ-15 and indeed IPTS theory to the Chinese population. To this end, a series of exploratory factor analysis (EFA), confirmatory factor analysis (CFA), measurement methodology framework of the Item Response Theory (IRT), logistic regression analysis, and receiver operating characteristic (ROC) analyses were conducted. While the structure of the INQ has been confirmed on a solid theoretical basis by Van Orden et al. [14], we believe that it is crucial to validate the translated version in the Chinese population and that the results may vary. Following factor analysis, we examined the psychometric properties of the TB and PB and their related constructs by comparing them and examining whether they accurately predicted suicidal ideation or whether one of the constructs had better predictive power.

## 2. Method

### 2.1. Participants and Procedures

A cross-sectional survey of industrial workers in Shenzhen (China’s largest migrant city) was conducted from October to December 2019. A stratified multi-stage sampling method was used for recruitment from 16 factories and 513,215 industrial workers.

Assuming a prevalence of past-year suicidal ideation in migrant workers of 15% [18,33,34], using α of 0.05, and a relative error for sampling of 0.1, we calculated a required sample size of 1533 to allow for a non-response rate of 50%.

First, 16 factories, including 4 machinery processing factories, 3 electronic device manufacturing factories, 3 printing and dyeing factories, 2 chemical material factories, 1 smelting factory, 1 garment factory, 1 food and beverage manufacturing factory, and 1 other factory were randomly selected. Full-time employees aged ≥18 years in 3 to 4 workshops from each factory were then randomly invited to participate in a survey conducted at the Longhua District Center for Disease Control and Prevention (CDC).

Each participant signed a written informed consent and was informed of the potential benefits and risks of participating by trained fieldworkers before the survey. Since the questionnaire contained questions about their mental health, the researcher provided respondents with assurance of anonymity during the survey, the right to quit at any time, and that there would be no consequences for refusing the survey. Each participant who spent approximately 30 min to complete a survey in a private room would be compensated with a USD 2.60 cash voucher.

A group of two public health researchers, an epidemiologist, a health psychologist, a health communication expert, and a factory worker designed the questionnaire. Twenty workers were invited to participate in a pilot study under the instruction of trained researchers. Some reading and comprehension problems were recorded in the process and the questionnaire was revised and finalized based on their feedback. These 20 workers did not participate in the final survey.

### 2.2. Instruments and Measures

The study collected sociodemographic information on workers’ age, gender, place of origin, monthly income, living alone or not, and time staying in Shenzhen, and used the following self-reporting tools.

#### 2.2.1. Chinese Version of the Interpersonal Needs Questionnaire-15 (INQ-15)

The 15-item INQ, a self-report scale, published by Van Orden K.A. [14] and translated by Cao [35], was used to measure thwarted belongingness and perceived burdensomeness. Previous research on the two-dimensional INQ-15 scale has shown that the first six items measure perceived burdensomeness and the last nine items measure thwarted belongingness. Participants were asked to indicate how true each item had recently become for them. A 7-point Likert scale was used, ranging from “not true for me at all” to “very true for me”. Six items on the TB scale were reverse scored and total scores were coded, with higher scores reflecting greater perceived burdensomeness or thwarted belonging. The INQ-15 has been shown to predict suicidal ideation [13]. The subscales have demonstrated good internal consistency, with Cronbach’s alpha values ranging from 0.85 to 0.90 for PB, and 0.81 to 0.87 for TB [39,40].

#### 2.2.2. The Suicidal Ideation Scale

The suicidal ideation scale consists of three questions: “Have you considered suicide in the past year?”; “Have you planned suicide in the past year?”; and “Have you committed or almost committed suicide in the past year?”. In this study, anyone who answered “yes” to any of these three questions was considered to be at risk of suicide. A previous study has shown that single-item assessments of suicidal ideation are significantly associated with total suicidal ideation scores from multiple assessments [41].

### 2.3. Ethics Approval

The study was conducted according to the guidelines of the Declaration of Helsinki and approved by the Ethics Committee of School of Public Health, Sun Yat-sen University (2019/3).

## 3. Data Analysis

All completed questionnaires were recorded by two people via Epidata (The EpiData Association, Odense, Denmark). Means and standard deviations are reported for normally distributed data. A review of kurtosis and skewness indicated that INQ-15 scores were not normally distributed; therefore, non-parametric tests and medians (interquartile range; IQR) are reported throughout the analysis. Exploratory factor analysis and confirmatory factor analysis were conducted using Mplus 8 (Muthén & Muthén, Los Angeles, CA, USA). Robust maximum likelihood (MLR) estimators robust to non-normal data [42] and oblique rotations allowing for correlation of the examined factors [42] were applied. The IRT allows one to maintain only those items that contribute unique variance in the measured construct, making it possible to provide more precise psychometric understanding of each item’s contribution to an overall mean score [43]. Data screening and regression analyses were performed using SPSS version 25 (IBM, Armonk, New York, NY, USA). To assess the accuracy of the INQ-15 total score (sum of items) and subscale scores in predicting suicidal ideation, receiver operating characteristic (ROC) analyses were conducted.

Several fit indices were considered, since each index of model fit has unique properties that give rise to strengths and weaknesses: two absolute fit indices, the chi-square (χ^2^) and the standardized root-mean-square residual (SRMR); two comparative fit indices, the comparative fit index (CFI) and the Tucker–Lewis index (TLI); and a parsimonious-corrected fit index for root-mean-square error of approximation (RMSEA).

All fit statistics were associated with the MLR estimator of Mplus, and the reported chi-square was the Yuan–Bentler chi-square [41]. CFI and TLI values > 0.90 and RMSEA and SRMR values < 0.08 were identified as acceptable model fit metrics in this study [44,45].

## 4. Results

**Figure 1:**
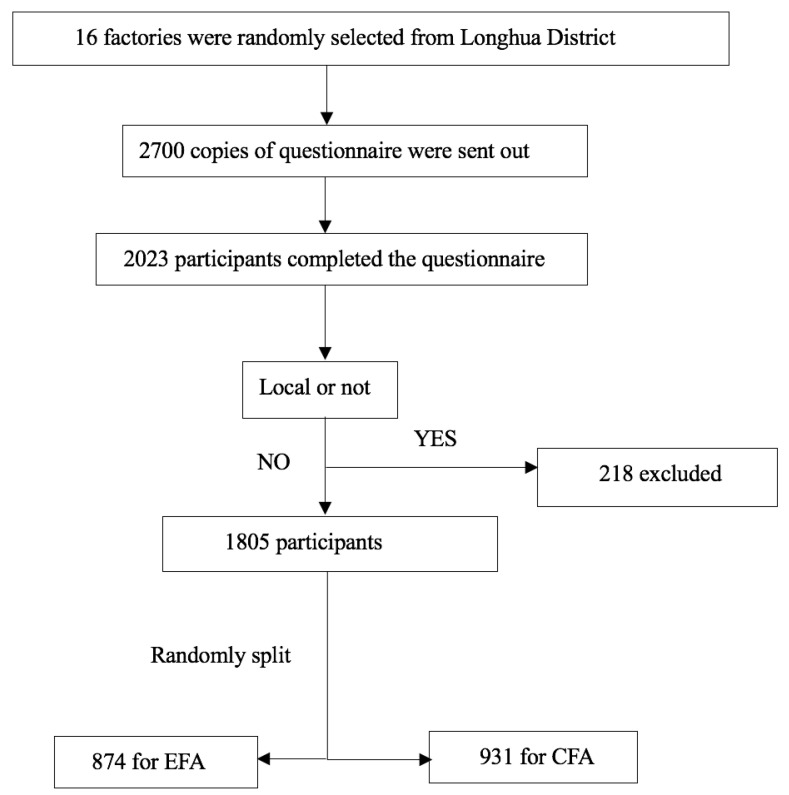
Participants recruitment flow chart.

Of 2700 workers selected, 2023 completed a self-administered questionnaire. The response rate was 75%. Since we focused on migrant workers, local workers and those who did not report their homeland were excluded. Eventually 1805 migrant workers were included, as shown in Figure 1. The participant age range was from 18 to 61, the mean age was 32.50 (SD: 8.43), and 67.3% of them were male. All participants were randomly split in two groups with equal numbers. The first group including 874 workers was used for exploratory factor analysis. The mean age was 32.43 (SD: 8.41) and 67.6% of them were male. The confirmatory factor analysis was conducted on the second group including 931 workers with a mean age of 32.56 (SD: 8.44) who were 66.9% male.

**Figure 2:**
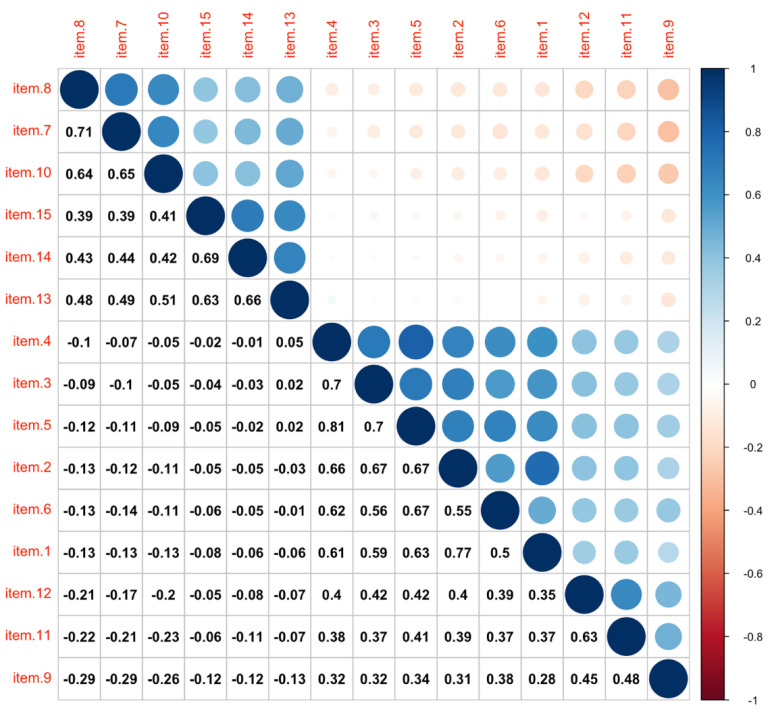
Intercorrelations among INQ Items. (*n* = 1805).

Descriptive statistics and intercorrelations of the INQ items for all participants are presented in Table 1 and Figure 2. The result of the Shapiro–Wilk test (*p* < 0.001 for all 15 INQ items) suggested that responses of the 15 INQ items were not normally distributed. Therefore, estimators of robust to non-normality were used.

## 5. Factor Structure of the Interpersonal Need Questionnaire

### 5.1. The Exploratory Factor Analysis

As expected, multiple items of the INQ were skewed positively, implying that the INQ measured non-normally distributed. Most items especially those of the same factor were significantly correlated, supporting appropriate internal consistencies, except for item 1/2 with item 13, item 3/4 with item 14 and item 15, and item 5 with item 13.

The results of the exploratory factor analysis 1–4, as displayed in Table 2, showed that the three-factor and four-factor model demonstrated better model fits. However, the factor loading of a three-factor model had better indicators for each item than four-factor model. The pattern of loadings for this model in Sample 1 indicated that the first six items and items 9/11/12 clearly loaded onto a “PB” factor, which means the three items written to measure thwarted belongingness cross-loaded onto perceived burdensomeness. Items 7/8/10 loaded onto thwarted belongingness, a “TB” factor, and the last three items strongly loaded on a third factor, which we named “social isolation”, an “SI” factor. The oblique rotated loadings for three-factor model are showed in Table 3.

### 5.2. The Confirmatory Factor Analysis

Fit statistics for the CFA in Sample 2 are presented in Table 2. The fit statistics for original three-factor model in Sample 2 did not meet the acceptable criteria proposed before. Therefore, the model was refined according to the modification index. The inclusion of residual correlations between the items 1 and 2 and between the items 11 and 12 improved the fit of the model clearly, as shown in Table 3. The values of both RMSEA and SRMR were below 0.08. In addition, the CFI and TLI values exceeded the cutoff score of 0.90, supporting a good model fit. The χ^2^ value was still significant and the χ^2^/df value was still higher than acceptable level. However, the χ^2^ value has been constantly criticized for its sensitivity to sample size [46] as the χ^2^ value will always be significant, resulting in the rejection of the model fit in large samples. Due to the controversy, the χ^2^ value was given less attention when it conflicted with other model fit indices.

Estimated parameters for the refined model (standardized estimated factor loadings SE, covariances, and R^2^ values) are presented in Table 4. All items loaded onto the anticipated latent variable significantly with R^2^ ranging from 0.13 to 0.81. Along with the model fit indices, parameter estimates supported the three-factor model of the INQ-15. There was a positive correlation between the “SI” and the “TB” factor but negative correlations between the “PB” and “TB” or “SI” factor.

### 5.3. IRT Modeling and M2 Test

The RMSEA of the M2 test of the model was <0.001, which was far below 0.08, while the *p*-value was 0.74, indicating good model fit of the IRT modeling.

### 5.4. Predictive Validity of the INQ

**Figure 3:**
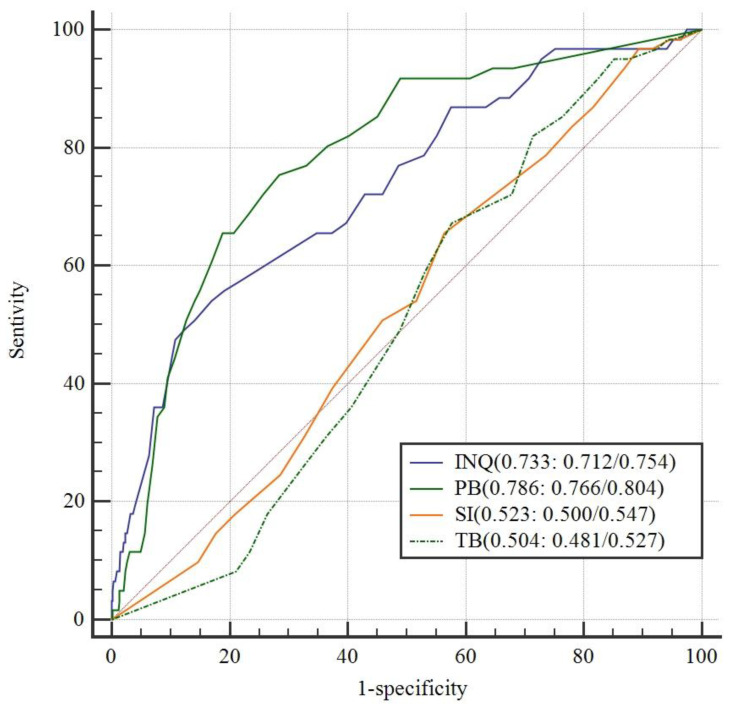
Receiver operator characteristic (ROC) curve for the Interpersonal Needs Questionnaire as a predictor of suicide ideation (*n* = 1805).

Logistic regression analysis (forward:LR) showed that the total scores of the INQ predicted suicidal ideation, with an OR = 1.084 (95% CI: 1.059/1.110), after controlling for sociodemographic characteristics. For our data, the self-reported rate of suicidal ideation was 3.4%, and using the INQ-15 summed item scores to assess suicidal ideation produced an AUC = 0.733 (95% CI: 0.712/0.754), as shown in Figure 3. Contrary to other studies [16,21] only the main effect of PB proved to be valid, with an OR = 1.094 (95% CI: 1.069/1.112) and with an AUC of 0.786 (95% CI: 0.766/0.804). In addition, the comparison of the predictive ability between the INQ total scores and PB scores was significant with *p* < 0.05. The cut-offs were 53 for the INQ score (sensitivity: 54.10; specificity: 82.97) and 18 for the PB score (sensitivity: 75.41; specificity: 71.62).

## 6. Discussion

This study aimed to validate the Chinese version of the Interpersonal Needs Questionnaire-15 among industrial workers and comprehensively test the association of interpersonal needs with suicidal ideation. Even though a growing number of studies have used the INQ to examine the applicability of the Interpersonal Psychological Theory of Suicide, as well as to examine its validity in different countries and various populations [17,22,24,26,27,29,38,39,47,48], the application of IPTS and INQ among Chinese people is rare [49,50]. Considering the collectivistic culture in China and the difference of INQ across cultures [51], we reasoned it was necessary to validate the INQ in a sample of industrial workers, whose suicide events caused great social attention in the past years.

The result of exploratory factor analysis showed a three-factor model demonstrated better model fit and factor loading, which is different from most existing studies [14,19,21,51]. As the INQ-15 was developed as an English language instrument for measuring interpersonal needs, cultural adaptation should be considered. Yeonsoo Park et. al.’s results implied that the reading of an item, and ultimately the experience of specific psychological states, can vary depending on cultural influence [16]. In a Slovene translation and validation study, the researchers chose to omit items loaded weakly on its hypothesized factor and reduced INQ 15 to INQ 12 [47]. However, Quintin A. Hunt et al. extracted a third factor of perceived isolation “PI”, formed from the TB factors instead of omitting them in a sample of clinically depressed and suicidal youth [27]. Meanwhile, it is also worth noting that most industrial workers in Shenzhen come from different cities and float around across the country. Most of their friends and social connections are in their hometown and people they work with are thus unfamiliar. So, it is possible that social isolation may exist if a specific population are removed from their living environment.

The CFA confirmed that perceived burdensomeness, thwarted belongingness, and social isolation are distinct but related, with thwarted belongingness and social isolation being closely related. The initial model demonstrated poor fit. However, when allowing two pairs of items to covary, the fit indices improved to excellent. Items 1 (i.e., “These days, the people in my life would be better off if I were gone”) and 2 (i.e., “These days, the people in my life would be happier without me”) refer to the positive effects of an individual’s death on others; items 11 (i.e., “These days, I feel disconnected from other people”) and 12 (i.e., “These days, I often feel like an outsider in social gatherings”) refer to the relationship between the self and outsiders. Because each pair shared a common theme, we reasoned that allowing the pair of items to covary would be acceptable just like other research [17,52]. The IRT modeling and M2 test approved the procedure. In our study, the TB was significantly negatively associated with PB and positively correlated with SI, and we interpreted such relationships to be due to the difficulty participants may have had dealing with reverse-coded questions.

In line with the interpersonal-psychological theory of suicidal behavior [12], the results of the current study suggest that the INQ strongly predicts desire for suicide. As socioeconomic factors including relative income [53,54] have been associated with suicidal behaviors, we re-ran a regression analysis to identify a unique contribution of INQ to suicidal ideation and the relation was still significant. Meanwhile, the effect of PB and TB was significant, before and after controlling for demographic characteristics. These results are in accordance with the theory, which assumes that all constructs are needed in order for suicidal ideation to develop [11,14], and the findings of some previous studies also support it [13,55]. Consistent with Mitchell et al. [56] and Jeffrey B. Brookings et al.’s findings, the results of the ROC analysis indicate that the INQ-15 may be useful and PB may be better in detecting suicidal ideation among individuals. Individuals whose INQ scores exceeded 53 or PB scores exceeded 18 may be at high risk of suicidal ideation and in need of help. In this study, a hotline number was given to all workers in case they needed psychological counseling. However, the INQ-15 was not developed as a screening tool. It was thus difficult to apply cut-off scores to measure suicidal ideation in reality.

Limitations of this study are that it focuses on migrant workers in Shenzhen only and that we did not repeat the survey to get the test-retest reliability; thus, the findings cannot be generalized to average populations in China. However, we did use a larger sample in our study, so the results may provide a worthy reference for the application of INQ-15 in large-sample population surveys. Second, since the culture effect has been demonstrated [16,51] and the Chinese version of INQ-15 was used in this study, the culture effect and translation influence were not tested. Third, the three questions measured short-time suicidal ideation rather than life-time suicidal ideation. However, the mean time the investigated workers were staying in Shenzhen was 6.24 years (SD: 6.34), so it was reasonable for the INQ to predict short-time suicidal ideation.

## 7. Conclusions

In conclusion, the INQ has good psychometric properties and can be used to assess how industrial workers living in the Shenzhen perceive meeting interpersonal psychological needs. To our knowledge, it is the first study to validate the Chinese version of INQ-15 in a sample consisting of migrant workers, and the validation of the scale among migrants is the first step necessary to test the interpersonal-psychological theory of suicide on a specific population. The translated INQ confirmed three distinguishable and internally consistent factors corresponding to PB, TB, and SI, and predicted suicidal ideation properly. Future studies may seek to develop norms or cut-off scores at which elevated scores on the INQ-Chinese would indicate an elevated risk for suicide and focus on interventions in this process to prevent suicide.

## Figures and Tables

**Table 1:** Sociodemographic characteristics of industrial workers. (*n* = 1805).

Sociodemographic Characteristics	Number (%)
Gender	
Male	1214 (67.3)
Female	591 (32.7)
Education	
Less than high school	1059 (58.6)
High school	585 (32.4)
College degree or above	113 (6.3)
Unknown	48 (2.7)
Income (RMB)	
<3000	166 (9.2)
3000–4999	1099 (60.9)
>4999	484 (26.8)
Unknown	56 (3.1)
Staying time in Shenzhen (years)	
<10	1471 (81.5)
10–20	231 (12.8)
>20	103 (5.7)
Living with others	
Relatives	944 (52.3)
Living alone	592 (32.8)
Unknown	269 (14.9)
Suicidal ideation	
Yes	61 (3.4)
No	1744 (96.6)

**Table 2:** Fit statistics for EFA/CFA models.

	Model	χ^2^	df	*p*-Value	RMSEA	90% CI	CFI	TLI	SRMR
EFA	one-factor	2253.788	90	<0.001	0.166	0.160–0.172	0.495	0.411	0.188
	two-factor	869.495	76	<0.001	0.109	0.103–0.116	0.815	0.744	0.060 ^a^
	three-factor	511.126	63	<0.001	0.090	0.083–0.098	0.895	0.826	0.041 ^a^
	four-factor	323.748	51	<0.001	0.078 ^a^	0.070–0.086	0.936 ^a^	0.869	0.025 ^a^
CFA	three-factor	917.033	87	<0.001	0.101	0.095–0.107	0.897	0.876	0.074 ^a^
	three-factor refined	529.394	85	<0.001	0.075 ^a^	0.069–0.081	0.945 ^a^	0.932 ^a^	0.067 ^a^

^a^ Fit indicator is within acceptable limits. Three-factor refined = INQ-15 with covariances.

**Table 3:** Oblique rotated loadings for three-factor model for Sample 1 (*n* = 874).

Item	Description	PB	TB	SI
Item 1	These days the people in my life would be better off if I were gone	0.727 *		
Item 2	These days the people in my life would be happier without me	0.798 *		
Item 3	These days I think I am a burden on society	0.789 *		
Item 4	These days I think my death would be a relief to the people in my life	0.875 *		
Item 5	These days I think the people in my life wish they could be rid of me	0.880 *		
Item 6	These days I think I make things worse for the people in my life	0.725 *		
Item 7	These days, other people care about me		0.639 *	
Item 8	These days, I feel like I belong		0.615 *	
Item 9	These days, I rarely interact with people who care about me	0.400 *		
Item 10	These days, I am fortunate to have many caring and supportive friends		0.549 *	
Item 11	These days, I feel disconnected from other people	0.496 *		
Item 12	These days, I often feel like an outsider in social gatherings	0.483 *		
Item 13	These days, I feel that there are people I can turn to in times of need			0.757 *
Item 14	These days, I am close to other people			0.829 *
Item 15	These days, I have at least one satisfying interaction every day			0.855 *

* significant at 5% level.

**Table 4:** Model estimated factor loadings, covariances, *p*-value and R-squares in Sample 2 (*n* = 931).

	Estimated	S.E.	*p*	R-sq	Item-Total Correlation
Perceived Burdensomeness					
Item 1	0.730	0.017	<0.001	0.532	0.452 *
Item 2	0.781	0.014	<0.001	0.610	0.497 *
Item 3	0.821	0.012	<0.001	0.674	0.566 *
Item 4	0.869	0.010	<0.001	0.755	0.589 *
Item 5	0.901	0.008	<0.001	0.811	0.565 *
Item 6	0.713	0.018	<0.001	0.508	0.511 *
Item 9	0.366	0.030	<0.001	0.134	0.260 *
Item 11	0.430	0.028	<0.001	0.185	0.334 *
Item 12	0.471	0.027	<0.001	0.222	0.391 *
Thwarted Belongingness					
Item 7	0.859	0.013	<0.001	0.738	0.467 *
Item 8	0.842	0.014	<0.001	0.709	0.436 *
Item 10	0.774	0.016	<0.001	0.600	0.447 *
Social Isolation					0.613 *
Item 13	0.806	0.016	<0.001	0.649	0.605 *
Item 14	0.836	0.015	<0.001	0.699	0.502 *
Item 15	0.802	0.016	<0.001	0.643	0.496 *
Covariances					
Item 1 with Item 2	0.387	0.031	<0.001		
Item 11 with Item 12	0.504	0.025	<0.001		
TB with PB	−0.168 *	0.036	<0.001		
SI with PB	−0.055	0.037	0.136		
SI with TB	0.651 *	0.025	<0.001		

* significant at 5% level.

## Data Availability

Data are available on request due to privacy and ethical restrictions. The data presented in this study are available on request from the corresponding author. The data are not publicly available due to protection of participants’ privacy.
